# Composition, Structure and Functional Properties of Protein Concentrates and Isolates Produced from Walnut (*Juglans regia* L.)

**DOI:** 10.3390/ijms13021561

**Published:** 2012-02-02

**Authors:** Xiaoying Mao, Yufei Hua

**Affiliations:** 1 State Key Laboratory of Food Science and Technology, School of Food Science and Technology, Jiangnan University, 1800 Lihu Avenue, Wuxi, 214122, Jiangsu Province, China; E-Mail: maoxiaoying99@163.com; 2 Food College of Shihezi University, Shihezi, 832003, Xinjiang Province, China

**Keywords:** walnut protein concentrate, walnut protein isolate, composition, structure, functional properties

## Abstract

In this study, composition, structure and the functional properties of protein concentrate (WPC) and protein isolate (WPI) produced from defatted walnut flour (DFWF) were investigated. The results showed that the composition and structure of walnut protein concentrate (WPC) and walnut protein isolate (WPI) were significantly different. The molecular weight distribution of WPI was uniform and the protein composition of DFWF and WPC was complex with the protein aggregation. *H*_0_ of WPC was significantly higher (*p* < 0.05) than those of DFWF and WPI, whilst WPI had a higher *H*_0_ compared to DFWF. The secondary structure of WPI was similar to WPC. WPI showed big flaky plate like structures; whereas WPC appeared as a small flaky and more compact structure. The most functional properties of WPI were better than WPC. In comparing most functional properties of WPI and WPC with soybean protein concentrate and isolate, WPI and WPC showed higher fat absorption capacity (FAC). Emulsifying properties and foam properties of WPC and WPI in alkaline pH were comparable with that of soybean protein concentrate and isolate. Walnut protein concentrates and isolates can be considered as potential functional food ingredients.

## 1. Introduction

Plant proteins play significant roles in human nutrition, particularly in developing countries where average protein intake is less than that required. Plant protein products are gaining increased interest as ingredients in food systems throughout many parts of the world and the final success of utilizing plant proteins as additives depends greatly upon the flavor characteristics that they impart to foods. Since oilseeds are valuable sources of lipids as well as proteins, numerous studies on protein functionality of major and minor oilseeds such as soybean [1], peanut [2], rapeseed [3], sunflower [4], almond [5], winged bean [6], ground nut [7], have been reported.

Walnuts (*Juglans regia* L.) are widely distributed all over the world, and they are common in China. On a global basis, walnuts rank second behind almonds in tree nut production. In 2010, global production of walnuts was 1,500,000 t. China leads the world production of walnuts, followed by the US. In 2010, China accounted for 33.33% of global walnut production. Walnuts are receiving increasing interest as a healthy foodstuff because their regular consumption has been reported to decrease the risk of coronary heart disease [8,9]. Therefore, walnuts can be utilized as ingredients of many foodstuffs such as bakery products to enhance the nutrition value and sensory properties of the final product [10]. Walnuts oil is a major product of walnut production and is one of the important special oils used for salad dressing and cooking [11]. Other proposed benefits of walnuts include their high content of protein, magnesium, copper, folic acid, potassium, fiber and vitamin E [12]. Walnuts are a good source of high quality protein and contain 18~24% protein on a dry weight basis (dwb) [13]. As a by-product of oil production, walnut protein products are therefore being considered as an additional source of plant protein for use in human food products. Walnuts nutrient composition has been investigated by several investigators [13–16]. However, functional properties of walnut protein have not been investigated. Therefore, the objective of this study was to determine the composition, structure and the functional properties of defatted walnut flour, walnut protein concentrate and walnut protein isolate. The investigated functional properties included the water solubility, emulsifying activity, emulsifying stability, foaming capacity and foam stability, fat absorption capacity, and water absorption capacity.

## 2. Results and Discussion

### 2.1. Chemical Characterization of Defatted Walnut Flour and Walnut Protein Concentrate and Isolate

The proximate composition of defatted walnut flour (DFWF), walnut protein concentrate (WPC) and walnut protein isolate (WPI) is shown in Table 1. DFWF contained 1.80% fat. It was indicated that defatted procedure could reduce the fat content of samples effectively. DFWF was used as starting material for the preparation of WPC and WPI. Protein content of DFWF, WPC and WPI was significantly different (*p* < 0.05), with value of 52.51%, 75.56%, 90.50%, respectively. The difference in protein content for WPC and WPI may be attributed to the extraction method used. The yield of WPI and WPC was 43.15% and 76.60%. Results indicated that the alkaline extraction-isoelectric precipitation method can improve the protein content of walnut protein products better than isoelectric precipitation process. It was suggested that walnut protein concentrate and walnut protein isolate could be considered as an additional source of plant protein in food products.

### 2.2. Amino Acid Analysis

Amino acid composition is an important chemical property of proteins, as it determines their nutritional value. Amino acid compositions of DFWF, WPI and WPC are presented in Table 2. When comparing the amino acid composition of DFWF, WPI and WPC with the FAO/WHO [17] recommended pattern, it appeared that the DFWF essential amino acids content was higher than the values recommended for a pre-school child (2~5 years old), except for lysine, phenylalanine and methionine. It also appeared that lysine was the first limiting amino acid in both walnut protein products, with values of 2.01 g/100 g protein (WPC) and 2.13 g/100 g protein (WPI), respectively, which was in accordance with cashew nut, pine nut and hazelnut [17]. DFWF lysine and phenylalanine content was higher than that of WPI, which was also higher than that of WPC. This was the consequences of alkaline treatments and isoelectric precipitation, as these can cause chemical modifications of some amino acid residues [18]. In addition, DFWF, WPC and WPI contained high level of isoleucine, with value of 3.28 g/100 g protein, 3.03 g/100 g protein, 3.99 g/100 g protein, respectively. Compared to the FAO/WHO (1990) recommended pattern for adult, DFWF, WPC and WPI were only deficient in methionine. From these results we conclude that DFWF, WPC and WPI could be a good resource of essential amino acids for adults. Therefore, DFWF, WPC and WPI could be considered as a rich resource of vegetable protein as soy protein in human nutrition.

### 2.3. SDS-PAGE

**Figure 1:**
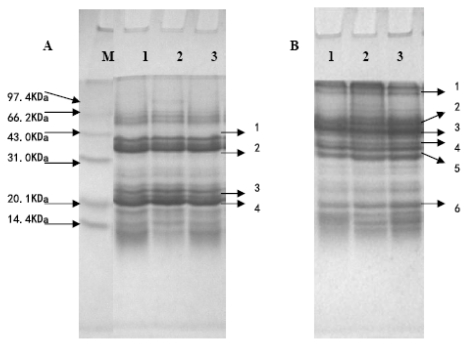
SDS-PAGE of DFWF, WPI and WPC: (**A**) Reduced (with β-mercaptoethanol); (**B**) Nonreduced (without β-mercaptoethanol); Marker (M), WPI (1), WPC (2), DFWF (3).

The non-reduced (without β-mercaptoethanol) and reduced (with β-mercaptoethanol) SDS-PAGE patterns for defatted walnut flour (DFWF), walnut protein isolate (WPI) and walnut protein concentrate (WPC) were presented in Figure 1. There was a noticeable difference on SDS-PAGE profiles between the non-reduced and reduced state. Following electrophoresis, DFWF, WPC and WPI showed more than 6 subunits at 110 KDa, 70 KDa, 55 KDa, 40 KDa, 35 KDa, 20 KDa (Figure 1B). Meanwhile, DFWF, WPC and WPI showed 4 polypeptides at 40 KDa, 35 KDa, 23 KDa, 20 KDa (Figure 1A). The results were similar to the reports by Sze-Tao and Sathe [13]. In contrast, the three major bands with molecular weight at 40 KDa, 35 KDa, 20 KDa were not affected by the presence of β-mercaptoethanol. Moreover, SDS-PAGE profiles showed no major qualitative differences among DFWF, WPC and WPI, which indicated that alkaline extraction-isoelectric precipitation technique and isoelectric precipitation technique did not change the protein composition. Also, this result suggested that bonded disulfide was associated with walnut protein and this may be due to the low NSI of DFWF, WPC and WPI.

### 2.4. High Performance Size Exclusion Chromatography

**Figure 2:**
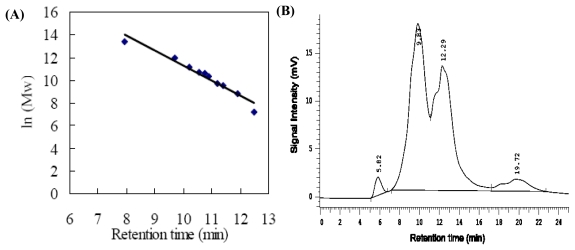

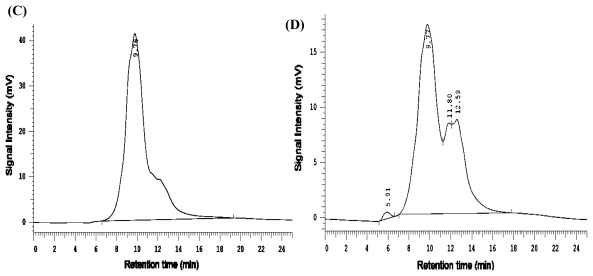
High performance size exclusion chromatography (SEC-HPLC) profiles of DFWF, WPC and WPI: (**A**) The calibration curve of standard proteins; (**B**) DFWF; (**C**) WPI; (**D**) WPC; A calibration curve of 10 standard proteins was used for interpreting the results. Ten standard proteins were thyroglobulin (MW: 669,000), aldolase (MW: 158,000), BSA (MW: 67,000), ovalbumin (MW: 43,000), peroxidase (MW: 40,200), adenylate kinase (MW: 32, 000), myoglobin (MW: 17,000), ribonuclease A (MW: 13,700), aprotinin (MW: 6500), and vitamin B12 (MW: 1350), respectively.

The size exclusion chromatogram using a high-performance liquid chromatogram system was used to study molecular weight distribution of walnut proteins and the results were shown in Figure 2. Molecular weight was estimated from the calibration curve of standard protein for the column (Figure 2A). DFWF showed four peaks with the retention time around 5.82 min, 9.83 min, 12.29 min, 19.72 min, corresponding to the molecular weight of 18,824 KDa, 96.99 KDa, 3.83 KDa (Figure 2B). The peak of 18,824 KDa may mark the protein aggregation of DFWF. WPI showed one peak with molecular weight of 106.33 KDa (Figure 2C). WPC showed four peaks with molecular weight of 16,725 KDa, 104.943 KDa, 7.3 KDa, 2.6 KDa, respectively (Figure 2D). There were minor differences in protein composition between DFWF, WPC and WPI. It was suggested that the protein composition of WPI was uniform and the protein composition of DFWF and WPC was complex with the protein aggregation. The results indicated that alkaline extraction-isoelectric precipitation technique could obtain uniform protein products without protein aggregation better than the isoelectric precipitation technique. However, the two peaks with the retention time around 9 min and 12 min were the main protein composition of DFWF, WPC and WPI. This result can explain why SDS-PAGE profiles showed no major qualitative differences among DFWF, WPC and WPI.

### 2.5. Circular Dichroism Spectra

**Figure 3:**
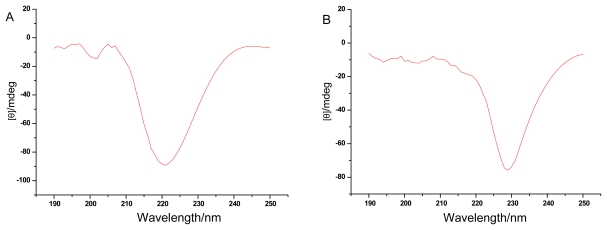

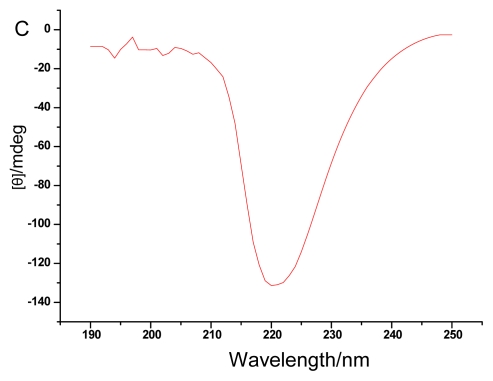
(**A**) Far-UV circular dichroism spectra of DFWF; (**B**) Far-UV circular dichroism spectra of WPI; (**C**) Far-UV circular dichroism spectra of WPC.

The CD spectra are remarkably sensitive to the secondary structures of proteins. Far-UV CD spectra of the DFWF, WPC and WPI were shown in Figure 3. Far-UV CD spectra of DFWF differed for WPI and WPC. DFWF was calculated composed of 80.4% α-helix, 4% β-sheet, 5.7% β-turn and 15.3% random coil by the computer program. The secondary structure estimation of WPI revealed 34.9% α-helix, 11% β-sheet, 23.3% β-turn and 32% random coil, which was similar to that of WPC. WPC and WPI contained more β-turn, random coil and less α-helix than DFWF, which suggested that WPC and WPI had lost its ordered secondary structure. The results indicated that alkaline extraction-isoelectric precipitation technique and isoelectric precipitation technique changed the secondary structure of walnut protein. This may be due to the higher NSI of WPI and WPC than in DFWF.

### 2.6. Surface Hydrophobicity

The elevated surface hydrophobicity (*H*_0_) value is indicative of high solubility, negligible aggregation, and prospect for exposure of hydrophobic components that are otherwise buried inside the globular structure of the protein due to denaturation. The surface hydrophobicity (*H*_0_) of protein concentrate, isolate and defatted flour of walnut were presented in Table 3. The results showed that *H*_0_ of WPC (315.39) in the present study was significantly higher (*p* < 0.05) than those of DFWF and WPI, whilst WPI had a higher *H*_0_ (276.51) compared to DFWF (223.83). Significant changes occurred on the *H*_0_ of walnut protein isolate and concentrate due to the isolation procedures. Meanwhile, isoelectric precipitation technique can increase the surface hydrophobicity of WPC better than that of WPI produced by alkaline extraction-isoelectric precipitation technique. The *H*_0_ of DFWF, WPC and WPI were higher than that of soy protein isolate (SPI) (206.76), reported by Ke-Xue Zhu *et al*. [19]. Generally, high *H*_0_ is considered a contributing factor to higher protein foaming capacity where this property is needed for a specific food product application [20].

### 2.7. Microstructure

**Figure 4:**
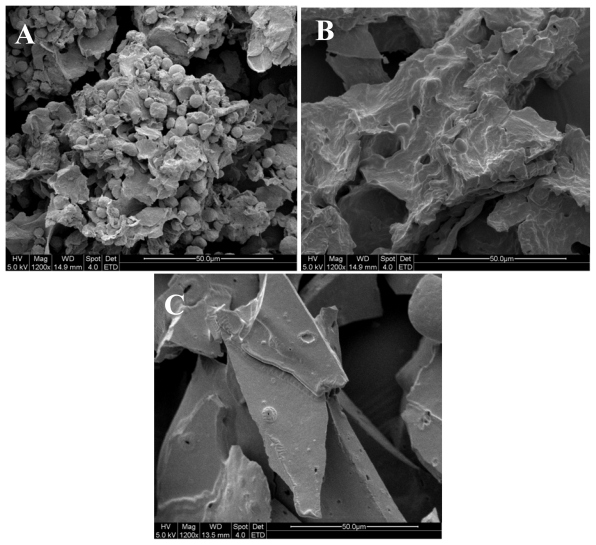
Scanning electron microscope pictures (1200× magnifications, bar 50 μm) of DFWF (**A**) WPC (**B**), WPI (**C**).

**Figure 5:**
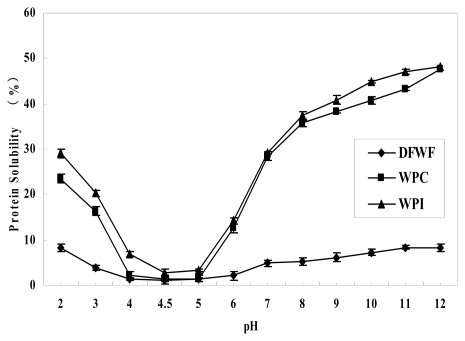
Effects of pH on solubility of DFWF, WPC, and WPI.

Structural morphology of the proteins was studied with the aid of scanning electron microscope (SEM). SEM pictures of defatted walnut flour (DFWF), walnut protein isolate (WPI) and walnut protein concentrate (WPC) (Figure 4) showed distinct surface structures. The WPI showed big flaky plate like structures; whereas WPC appeared as a small flaky and more compact structure. As DFWF consists of entire protein components, their morphological structure showed the mixture of fat particles and small flaky plate shapes. The results indicated that alkaline extraction-isoelectric precipitation technique and isoelectric precipitation technique changed the microstructure of walnut protein. It was suggested that the big flaky plate like structures of WPI further contributes to improve the solubility (Figure 5). The different microstructure of DFWF, WPC and WPI may contribute to the overall physiochemical and functional properties of walnut protein.

### 2.8. Protein Solubility

The protein pH-solubility profiles of DFWF, WPC and WPI were shown in Figure 5. The protein solubility of DFWF, WPC and WPI in water at different pH (2.0–12.0) showed the same U-shaped curves (Figure 5), similar to the protein solubility profiles reported for peanut proteins [2] and for cashew nuts [21]. Protein solubility of WPI, WPC and DFWF were significantly different (*p* < 0.05) and it was minimal for both samples in the range of 2.0–4.5 and then increased as the sample pH increased. Similar results were also observed in walnut protein as reported by Sze-Tao and Sathe [13]. DFWF, WPC and WPI presented minimum proteins solubility at pH 4.5 with values of 1.02%, 1.31% and 2.88%, respectively, and maximum protein solubility at pH 12 with values of 8.34%%, 47.54% and 48.23%, respectively. This helped to indicate the protein isoelectric point as, generally, solubility decreases as the pH increases until it reaches the isoelectric point and then increases. The loss of electrostatic repulsive forces provides beneficial conditions for the formation of protein aggregates; high bulk density and large diameter of the aggregates results in precipitation of protein [22]; then the protein solubility increases with further increase of pH. Electrostatic repulsive forces between the positively charged proteins help to keep them apart and increase protein-solvent interactions. Moreover, the protein solubility of WPC and WPI were significantly higher (*p* < 0.05) than that of DFWF at all pH; these results were similar to the behavior observed in the protein solubility of defatted cashew nut powder, cashew nut protein concentrate and isolate reported by Semiu Olalekan Ogunwolu *et al*. [21]. However, the protein solubility of DFWF, WPC and WPI were lower than that of peanut protein isolate (60.5%), soy protein isolate (71.7%) as reported by Yu *et al*. [2], and Molina *et al*. [23], respectively. Walnut proteins were less soluble in water because they are mainly composed of glutelin [13]. In addition, the surface hydrophobicity contributed to the difference in walnut protein solubility. Solubility profile over a range of pH values is being used increasingly as a guide to protein functionality, since this relates directly to many important properties, e.g., use in beverages, emulsification, foaming capacity and gelation [24].

### 2.9. Emulsifying Properties

Emulsifying properties results of all the samples are presented in Table 3. At pH 7, the emulsifying activity (EA) of DFWF, WPC and WPI was significantly different (*p* < 0.05), with values of 53.28%, 52.98%, 50.01%, respectively. However, the emulsion stability (ES) of WPI was the highest (30.30%) and that of WPC was higher (27.45%) than that of DFWF (25.26%), being significantly different (*p* < 0.05). The results were in agreement with Chove *et al*. [25], who stated that the emulsifying capacity of proteins tends to decrease as protein concentration is increased and this was also consistent with the results reported on winged bean protein concentrate [26], sunflower protein isolate [27] and cashew nut protein concentrate and isolate [21].

**Figure 6:**
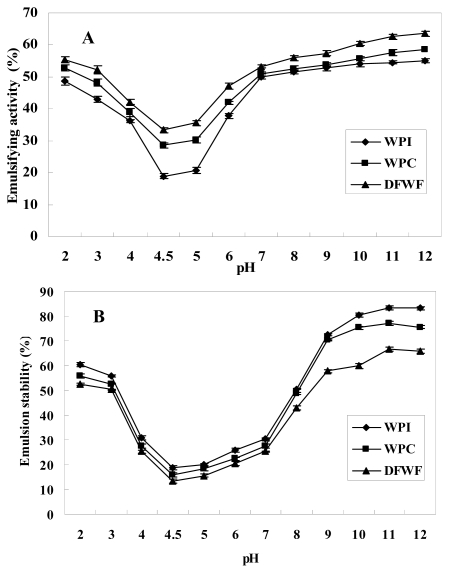
Effects of pH on emulsifying activity and emulsion stability of DFWF, WPC, and WPI: (**A**) emulsifying activity; (**B**) emulsion stability.

The effects of pH on the emulsifying activity (EA) of DFWF, WPC and WPI are shown in Figure 6A. The minimum emulsion activity of DFWF, WPC and WPI at pH 4.5 (isoelectric point) were 33.28%, 28.56% and 18.91%, respectively. A higher emulsifying activity was observed on both sides of the isoelectric point among DFWF, WPC and WPI. At pH 2, values of 55.45%, 52.78% and 48.61%, for DFWF, WPC and WPI, respectively, were found, and the highest emulsion activity was observed at pH 12; 63.62%, 58.45% and 55.12% for DFWF, WPC and WPI, respectively. The results indicated that emulsion capacity was pH-dependent and alkaline pH was found to improve the emulsion capacity more than acidic pH. The pH had pronounced effects on the emulsifying activity because soluble proteins emulsifying activity depend upon the hydrophilic-lipophilic balance [28], which in turn is affected by pH. At the oil–water interface, the protein oriented lipophilic residues to the oil phase and hydrophilic residue to the aqueous phase, thus reducing surface tension at the interface. At a pH of 4.5, protein solubility was low, protein adsorption at the oil–water interface would be diffusion controlled. However, at a pH range of 5.0–12.0 with protein solubility increased, the activation energy barrier did not allow protein migration to take place in a diffusion dependent manner. Increase in protein solubility facilitated enhanced interaction between the oil phase and the aqueous phase. The emulsifying activity of DFWF was higher than WPC and WPI in the tested pH range significantly (*p* < 0.05)*,* probably due to the difference in protein concentration. At low protein concentration, protein adsorption at the oil–water interface is diffusion controlled, since it will spread over the surface before it can be adsorbed. At high protein concentration, the activation energy barrier does not allow protein migration to take place in a diffusion-dependent manner [5]. Therefore, emulsifying activity decreased with increased protein concentration.

The effects of pH on emulsion stability (ES) of DFWF, WPC and WPI are shown in Figure 6B. The relationship between ES and pH for DFWF, WPC and WPI was similar to that of protein solubility and pH. The ES of all samples was low in the pH range 2.0–4.5, and then increased up to pH 11. In the pH range 7–9, the ES increased rapidly and afterwards it increased slowly up to pH 11. The highest emulsion stability (ES) among DFWF, WPC and WPI, was observed at pH 11 with values of 66.78%, 76.92% and 83.33%, respectively. This was in agreement with the correlation found between ES and nitrogen solubility in previous studies [29]. Another study has shown that the pH-emulsifying property profile of various proteins, including soy protein, resembles the pH-solubility profile [21]. The low ES at low pH might be attributed to increased interaction between the emulsified droplets, since net charge on the proteins was decreased by the presence of the chloride ions [30]. As the pH increased, the coulombic repulsion increased between neighboring droplets, coupled with increased hydration of the charged protein molecules. These factors resulted in reduction of interface energy and combination of emulsion droplet, which might account for the higher ES obtained [30]. The ES of WPI was significantly higher than that of WPC at all pH values (2–11), which in turn was significantly higher than that of DFWF. The differences in ES of DFWF, WPC and WPI might be due to their differences in protein content and the surface hydrophobicity of samples. An extensive protein–protein interaction, caused by hydrophobic interaction on the surface of the protein, would form a strong oil–water interface, resulting in a stable emulsion [31]. Comparisons between of EA and ES of walnut protein concentrate and isolate showed that the effects of pH on walnut proteins were greatly different, reflecting differences in their composition, solubility, structure, and interaction with other compounds and their surface hydrophobicity. High content of hydrophobic amino acids in protein improved its surface hydrophobicity and the exposed hydrophobic groups enhanced the interactions between proteins and lipids. The results indicated that emulsifying properties of walnut protein concentrate and isolate can be improved significantly in alkaline environment.

### 2.10. Foaming Properties

At pH 7, the foam capacity (FC) of WPI was significantly higher than that of WPC, which was significantly higher than that of DFWF, with the values of 46.34%, 38.78% and 24.35%, respectively. The foam stability (FS) of WPI was significantly higher than that of WPC, which was significantly higher than that of DFWF, with values of 30.56%, 28.18% and 10.23%, respectively (Table 3). The results suggested that the WPI had a more flexible protein structure in aqueous solutions and interacted strongly at the air–water interface to form more stable foams when compared to the WPC.

**Figure 7:**
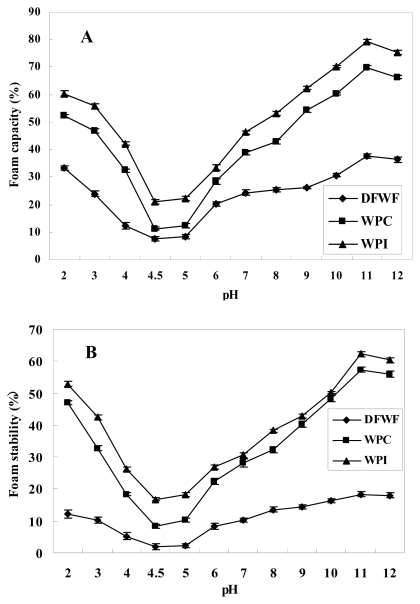
Effects of pH on foam capacity and foam stability of DFWF, WPC, and WPI: (**A**) foam capacity; (**B**) foam stability.

The effects of pH on the foam capacity (FC) and foam stability (FS) of DFWF, WPC and WPI are shown in Figure 7A,B. The lowest foam capacity and foam stability were observed at pH 4.5 (isoelectric point) among DFWF (7.39% and 1.98%), WPC (11.23% and 8.27%) and WPI (20.97% and 16.76%), respectively, which was also coincident with their lowest solubility (Figure 5) and lowest emulsifying properties (Figure 6). Beyond pH 4.5, FC and FS significantly increased for both samples, especially at pH 11. The results obtained were likely due to an increase in the net charge of the protein which weakens hydrophobic interaction and increases protein solubility and flexibility, allowing the protein to spread to the air–water interface more quickly, encapsulating air particles and thus increasing foam formation, as reported by Lawal *et al*. [32]. The profile of foaming properties against pH for the walnut protein isolate and concentrate was similar to that of its solubility against pH (Figure 5). Results revealed that the foaming properties of walnut protein isolate and concentrate were pH-dependent. Foaming properties improvement of walnut protein concentrate and isolate at alkaline pH may be due to increased solubility and surface activity of the soluble protein. Foaming properties of WPI was significantly higher (*p* < 0.05) than that of WPC at all pH values (2–11), which were in turn significantly higher (*p* < 0.05) than that of DFWF. The differences in foaming properties of both samples were likely due to their differences in protein concentration. The foam capacity and stability were enhanced by high protein concentration, as high protein concentration increases the viscosity and facilitates the formation of a multilayer, cohesive protein film at the interface [33].

### 2.11. Fat Absorption Capacity (FAC)

Fat absorption capacity (FAC) results of DFWF, WPC and WPI are presented in Table 3. Fat absorption capacity of both samples were significantly different (*p* < 0.05), with WPI having the highest FAC (2.81 g/g), followed by WPC (2.50 g/g) and DFWF (1.87 g/g) at pH 7. The differences in fat absorption capacity between the samples might be due to the presence of more non-polar amino acids in WPI and WPC than in DFWF, and also due to the partial denaturation of proteins with exposition of hydrophobic amino acids groups during the process of protein isolate production. The presence of several non-polar side chains may bind the hydrocarbon chains of fats, thereby resulting in higher absorption of oil [34]. El Nasri [35] reported that surface area and hydrophobicity improve fat absorption capacity and also high protein content shows high fat absorption capacity (FAC). Campell, Shih and Marshall [36] reported that FAC increased as protein content increased in sunflower and soy protein products. FAC values of WPI and WPC were higher than 2.434 g/g of commercial soy protein isolate (SPI) reported by Zhu *et al*. [19]. The ability of protein to bind fat is very important for applications as meat replacement and extenders, principally because it enhances flavor retention, and reputedly improves mouth feel [21]. Results obtained indicated that WPC and WPI had good oil absorption capacity. High FAC of WPI and WPC make them good ingredients in cold meat industry, particularly for sausages, where the protein usually bridge the fat and water in order to obtain good products.

### 2.12. Water Absorption Capacity (WAC)

Water absorption capacity (WAC) results of DFWF, WPC and WPI are presented in Table 3. Water absorption capacity of both samples were significantly different (*p* < 0.05), with DFWF having the highest WAC (3.57 g/g), followed by WPI (3.11 g/g) and WPC (2.94 g/g) at pH 7. However, the solubility of WPI was significantly higher than that of WPC and DFWF in Figure 5. It was suggested that there was no direct correlation between solubility and WAC of walnut proteins as high protein solubility did not necessarily mean high WAC. This was consistent with other studies findings [37]. Protein water absorption capacity of proteins is a function of several parameters, including size, shape, steric factors, conformational characteristics, hydrophilic-hydrophobic balance of amino acids in the protein molecules as well as lipids, carbohydrates and tannins associated with proteins. Carbohydrates contain hydrophilic parts, such as polar or charged side chains, which can enhance WAC [38]. DFWF water absorption capacity was enhanced, as the DFWF carbohydrate content (db, 43.94%) was significantly higher (*p* < 0.05) than that of WPI (db, 11.81%). WAC value of DFWF was higher than 3.551 g/g of commercial soy protein isolate (SPI) reported by Zhu *et al*. [19]. High WAC of DFWF makes it a potential ingredient for the meat, bread, and cakes industries.

## 3. Experimental Section

### 3.1. Materials and Methods

Walnuts (*Juglans regia* L.) were purchased from Xinjiang in China. The defatted walnut flour (DFWF) was produced according to the method suggested by Sze-Tao and Sathe [13] for the extraction of walnut protein. Walnut was ground in a Waring Blender. The flour was defatted with hexane (flour/hexane ratio of 1:10 w/v) under constant magnetic stirring for 3 h. The slurry was vacuum filtered through filter paper and the residue was used for subsequent extraction. Hexane extractions were repeated until the filtrate was clear. Residue from the last extraction and filtration step was air dried in a fume hood. DFWF was ground to 150 meshes with Waring Blender and stored at −20 °C for further use.

### 3.2. Preparation of Protein Concentrate

The walnut protein concentrate was prepared according to the process described by Wolf [39] with minor modifications. Defatted walnut flour with 95% aqueous alcohol (1:20, w/v) and stirred for 1h at ambient temperature (about 25 °C). The suspension was vacuum filtered through filter paper and the residues were air dried in a fume hood. Residues were dispersed in de-ionized water (1:20, w/v) at room temperature and the pH of the dispersion was adjusted to 4.5 by addition of 1NHCl, stirred using a magnetic stirrer for 2 h. The slurry was then centrifuged (10,000 g, 30 min, 4 °C) in a CR22G centrifuge (Hitachi Koki Co., Hitachinake, Japan). The precipitate was washed with de-ionized water, re-dissolved in water, neutralized to pH 7.0 with 1 N NaOH at room temperature, and then freeze-dried.

### 3.3. Preparation of Protein Isolate

The walnut protein isolate (WPI) was prepared according to the process described by Wolf [39] with minor modifications. DFWF was extracted by stirred for 2 h at room temperature (about 25 °C) with de-ionized water adjusted to pH 11.0 with 1 N NaOH (water: flour ratio, 1:20 (w/v)). The slurry was centrifuged at 10,000 g for 30 min at 4 °C. The insoluble walnut protein cake was re-dissolved with pH-adjusted de-ionized water as above, and cold-centrifuged again. The supernatants were mixed together, and were adjusted to pH 4.5 with 1 N HCl, and then kept for 2 h at room temperature (about 25 °C) and subsequently centrifuged at 10,000 g for 30 min at 4 °C. The precipitate was washed with water, re-dissolved in water, neutralized to pH 7 with 1 N NaOH at room temperature, and then freeze-dried.

### 3.4. Proximate Analysis

Moisture, fat and ash contents were determined according to the methods of AOAC [40], numbers 950.46, 960.39 and 920.153, respectively. The protein content of sample was determined by the micro-Kjeldhal method [41] through the use of the protein-nitrogen coefficient of 5.30 [13]. Carbohydrates were determined according to the method of Zhu *et al*. [42]. The contents were expressed on a dry weight basis. Each analysis was done in triplicate, and data were reported as means ± standard deviation.

### 3.5. Surface Hydrophobicity

Surface hydrophobicity (*H*_0_) was determined using 1-anilino-8-naphthalene sulphonate (ANS) as a fluorescence probe, as reported by Kato and Nakai [43]. Protein dispersions (4 mg/mL) were prepared in 0.01 M phosphate buffer (pH 7.0), stirred for 2 h at room temperature (about 25 °C). The protein concentration in the supernatant was determined by the Folin-phenol method [44]. Each supernatant was serially diluted with the same buffer to obtain protein concentrations ranging from 0.5 to 0.005 mg/mL. Then, a volume of 4 mL of each diluted sample was added with 50 μL of ANS (8.0 mM in 0.01 M, pH 7.0 phosphate buffer solution). Fluorescence intensity (FI) was measured with a Perkin-Elmer 2000 fluorescence spectrophotometer, at wavelengths of 365 nm (excitation) and 484 nm (emission). The net FI at each protein concentration was determined by subtracting FI of each solution without the probe from that with the probe. The initial slope of the FI *versus* protein concentration plot was used as an index of protein surface hydrophobicity (*H*_0_). Each analysis was done in triplicate, and data were reported as means ± standard deviation

### 3.6. Gel Electrophoresis

Protein subunits compositions were analyzed by sodium dodecyl sulphate polyacrylamide gel electrophoresis (SDS-PAGE). SDS-PAGE was performed according to the method of Laemmli [45] by the discontinuous buffer system at 4% stacking gel concentration and 12.5% separating gel concentration, using gel electrophoresis apparatus DYCZ-30 (Beijing Liuyi Instrument Factory, China). Electrophoresis was carried out in the presence and absence of β-mercaptoethanol (2% v/v) [46]. Samples were extracted with SDS-PAGE buffer for 4 h at room temperature (RT) using a vortex mixing. After heating in boiling water bath for 10 min, Samples were cooled to RT and then centrifuged (10,000 g, 10 min, RT). Supernatants were used for electrophoresis. The electrophoresis was run at 15 mA in the stacking gel and 25 mA in the separating gel until the tracking dye reached the bottom of the gel and gels were stained with Coomassie Brilliant Blue G 250. Subunit molecular weight was estimated by using a LMW calibration kit (Shanghai Institute of Biochemistry, China) consisting of hen egg white lysozyme (14.4 kDa), trypsin inhibitor (20.1 kDa), bovine carbonic anhydrase (31.0 kDa), rabbit actin (43.0 kDa), bovine serum albumin (66.2 kDa), and rabbit phosphorylase b (97.4 kDa).

### 3.7. Molecular Weight Distribution by SEC-HPLC

The molecular weight distribution was determined by High performance size exclusion chromatography (SEC-HPLC). Walnut protein concentrate, protein isolate and defatted walnut flour (5 mg ml^−1^) were extracted by sodium phosphate buffer (0.05 M, pH 8.0) containing sodium chloride (0.3 M) for 4 h at 25 °C under constant magnetic stirring and then were centrifuged at 10,000 g for 10 min (25 °C). The supernatant was filtered through a cellulose acetate membrane with a pore size of 0.45 μm (Sartorius Co, Ltd, Gottingen, Germany). A Waters 2690 liquid chromatogram system (Waters Chromatography Division, Milford, MA, USA) equipped with a Shodex protein KW-804 column (Shodex Separation and HPLC Group, Tokyo, Japan) and a Waters 996 photodiode array detector was used to determine the molecular weight distribution. The flow rate was 1 mL min^−1^ using phosphate buffer (0.05mol l^−1^, 0.3mol l^−1^ NaCl, pH 7.0) as the mobile phase. About 10 μL protein solutions were injected into the column and the eluent was monitored at 280 nm. All samples were measured in triplicate and the representative examples were selected for discussion. A calibration curve of 10 standard proteins was used for interpreting the results.

### 3.8. Amino Acid Analysis

Amino acids analysis was determined according to the method of Zhu, Zhou and Qian [42]. Samples of protein isolate and concentrate (100 mg) were subjected to acid hydrolysis with 5 mL of 6 M HCl under nitrogen atmosphere for 24 h at 110 °C. Each hydrolysate was washed into a 50 mL volumetric flask and made up to the mark with distilled water. The amino acids were subjected to RP-HPLC analysis (Agilent 1100) after precolumn derivatisation with *O*-phthaldialdehyde (OPA) or with 9-fluorenylmethyl chloroformate (FMOC). Methionine and cysteine were determined separately by oxidation products before hydrolysis in 6 M HCl. Amino acid composition was reported as g of amino acid/100 g of protein.

### 3.9. Circular Dichroism (CD) Spectra Measurement

CD spectra were scanned at the far-UV range (200–250 nm) with a CD spectropolarimeter (Jasco J-715, Jasco Corp., Tokyo, Japan) in a 0.1 cm quartz CD cuvette (Hellma, Muellheim, Baden, Germany) at 25 °C. The protein concentration for CD analysis was 50 mg/mL. Distilled water used to dissolve walnut protein samples was used as blank solution for all of the samples. The values of scan rate, response, bandwidth, and step resolution were 100 nm/min, 0.25 s, 1.0 nm, and 0.2 nm, respectively. Five scans were averaged to obtain one spectrum. The CD data were expressed in terms of mean molar ellipticity [θ] (deg cm^2^ dmol^−1^).

### 3.10. Scanning Electron Microscopy (SEM) Analysis

In order to investigate the influence of extracted techniques on the structure of the materials and to understand the extraction mechanism, the walnut protein samples were dried in air for the SEM analysis. Sample particles were fixed on the silicon wafer and sputtered with gold to a thickness of about 100 nm. The shape and the surface characters of the samples were observed and recorded on the scanning electron microscope (Quanta-200, FEI Ltd., Holand).

### 3.11. Water Protein Solubility

This was determined according to the modified methods of Rodriguez-Ambriz *et al*. [47]. 200 mg of sample were dispersed in 20 mL of de-ionized water and pH of the mixture was adjusted to 2, 3, 4, 4.5, 5, 6,7, 8, 9, 10, 11, 12 with 1 N HCl and 1 N NaOH. The mixture was stirred at room temperature for 30 min and centrifuged at 8000 g for 20 min. Protein contents in the supernatant were determined using the Bradford method [48]. All analysis was performed in triplicate. Protein solubility was then calculated by:

$$\text{Solubility }\%=\frac{\text{protein content in supernatant}}{\text{total protein content in sample}}\times 100$$

### 3.12. Emulsifying Properties

Emulsifying activity (EA) was determined according to the method of described by Pedroche *et al*. [49] with some modifications. Samples (1.0 g) were homogenized at a speed of 10,000 rpm for 1 min at room temperature (about 25 °C) in 25 mL de-ionized water. Then the pH of the solution was adjusted to 2, 3, 4, 4.5, 5, 6, 7, 8, 9, 10, 11, 12 with either 0.1 M HCl or 0.1 M NaOH. The protein solution was mixed with 25 mL of soybean oil followed by homogenization at a speed of 10,000 rpm for 1 min. Finally, the emulsion was centrifuged at 1300 g for 5 min. All analysis was performed in triplicate.

Emulsifying activity was determined by:

$$\text{Emulsifying activity }\%=\frac{\text{Height of emulsified layer}}{\text{Height of the contents of the tube}}\times 100$$

Emulsion stability (ES) was measured by re-centrifugation followed by heating at 80 °C for 30 min and then expressed as follows:

$$\text{Emulsion stability }\%=\frac{\text{Height of remaining emulsion layer}}{\text{Height of original emulsified layer}}\times 100$$

### 3.13. Foaming Capacity (FC) and Foam Stability (FS)

Foaming capacity (FC) and stability (FS) were based on the method described by Sze-Tao and Sathe [5] with minor modifications. 500 mg samples were dispersed in 50 mL of de-ionized water. The pH of the protein solution was adjusted to 2, 3, 4, 4.5, 5, 6, 7, 8, 9, 10, 11, 12 with either 0.1 M HCl or 0.1 M NaOH. The solutions were stirred at a speed of 10,000 rpm for 2 min. The blend was immediately transferred into a 100 mL graduated cylinder. The volume was recorded before and after stirring. FC was expressed as the volume (%) increased due to stirring. For the determination of FS, foam volume changes in the graduated cylinder were recorded at 30 min of storage. All analysis was performed in triplicate. Foam capacity and foam stability were then calculated according to the following formulae:

$$\begin{array}{c} \text{Foam capacity }\%=\frac{(\text{volume after whipping}-\text{volume before whipping})\text{ml}}{(\text{volume before whipping})\text{ml}}\times 100\\ \text{Foam stability }\%=\frac{(\text{volume after standing}-\text{volume before whipping})\text{ml}}{(\text{volume before whipping})\text{ml}}\times 100 \end{array}$$

### 3.14. Fat Absorption Capacity (FAC)

Fat absorption capacity was determined using the method described by Lin and Zayas [50] with minor modifications. 1 g of sample was weighed into 15 mL pre-weighed centrifuge tube and thoroughly mixed with 5 mL soybean oil. The emulsion was incubated at room temperature (about 25 °C) for 30 min and then centrifuged at 5000 g for 20 min at 25 °C. Then the supernatant was carefully removed, and the tube was reweighed. All analysis was performed in triplicate. FAC (gram of oil per gram of protein) was determined by:

$$\text{FAC}=\frac{{\text{F}}_{2}-{\text{F}}_{1}}{{\text{F}}_{0}}$$

where *F*_0_ is the weight of the dry sample (in gr), *F*_1_ is the weight of the tube plus the dry sample (in gr), and *F*_2_ is the weight of the tube plus the sediment (in gr).

### 3.15. Water Absorption Capacity (WAC)

Water absorption capacity was determined using the method described by Rodriguez-Ambriz *et al*. [47] with minor modifications. 1 g of sample was weighed into 15 mL pre-weighed centrifuge tube. Then 10 mL of distilled water was added in small increments to the tube under continuous stirring with a glass rod. After being held at room temperature (about 25 °C) for 30 min, the tube was centrifuged at 2000 g for 20 min. In the end, the amount of added distilled water resulting in the supernatant liquid in the test tube was recorded. All analysis was performed in triplicate. WAC expressed as grams of water per gram of sample, was calculated by:

$$\text{WAC}=\frac{{\text{W}}_{2}-{\text{W}}_{1}}{{\text{W}}_{0}}$$

where *W*_0_ is the weight of the dry sample (in gr), *W*_1_ is the weight of the tube plus the dry sample (in gr), and *W*_2_ is the weight of the tube plus the sediment (in gr).

### 3.16. Statistical Analysis

All analyses were done in triplicate, and data were reported as means ± standard deviation. Where appropriate, data were analyzed for significance using analysis of variance and Fisher’s least significant difference (LSD at a 5% significance level) by General Linear Model of SPSS (Software version 11.0, SPSS, Chicago, Illinois).

## 4. Conclusions

Results from this study indicate that the composition and structure of walnut protein concentrates and isolates were different when compared with defatted walnut flour. Also, functional properties of protein products were improved when walnut protein isolates and concentrates were produced from defatted walnut flour by isolation techniques. Furthermore, walnut protein isolates and concentrates could be good resources of essential amino acids for adults except methionine, and could be considered as a rich resource of vegetable proteins similar to other oilseed proteins in human nutrition. Finally, walnut protein isolate and walnut protein concentrate exhibit satisfactory functional properties as required in meat, cake and ice cream products processing, and as a good source of a protein ingredient in food systems. The production of walnut protein concentrate and isolate could also add value to defatted walnut flour, a low value by-product of walnut oil production.

## Figures and Tables

**Table 1:** Chemical composition of defatted walnut flour (DFWF), walnut protein concentrate (WPC) and walnut protein isolate (WPI) a.

Materials	Protein (%)	Fat (%)	Ash (%)	Moisture (%)	Carbohydrate (%)	NSI b (%)	Yield (%)
DFWF	52.51 ± 0.33c	1.80 ± 0.12a	1.94 ± 0.02	9.20 ± 0.02a	34.55 ± 0.16a	4.85 ± 0.12c	
WPI	90.5 ± 0.45a	0.23 ± 0.04b	2.27 ± 0.03	4.50 ± 0.03b	2.50 ± 0.03c	29.06 ± 0.23a	43.15
WPC	75.56 ± 0.35b	0.27 ± 0.06b	2.04 ± 0.02	4.48 ± 0.04b	17.65 ± 0.07b	28.24 ± 0.18b	76.60

a Results represent the average of three determinations ± SD, values in the same column with different letters are significantly different (*p* < 0.05);

b NSI, Nitrogen solubility index.

**Table 2:** Amino acid composition a of DFWF, WPC and WPI b.

Amino acids	DFWF	WPC	WPI	*P*-values	FAO/WHO c (1990)
Asp	10.04 ± 0.21a	6.95 ± 0.19c	9.38 ± 0.11b	0.00572	
Glu	22.16 ± 0.12a	15.78 ± 0.20c	19.49 ± 0.11b	<0.0001	
Ser	5.84 ± 0.19a	3.96 ± 0.22c	5.15 ± 0.07b	0.01093	
His	2.38 ± 0.13a	1.89 ± 0.09b	2.30 ± 0.10a	0.03921	1.9 (1.6)
Gly	5.43 ± 0.15a	3.54 ± 0.11c	4.18 ± 0.08b	0.02960	
Thr	3.58 ± 0.21a	2.55 ± 0.09b	3.30 ± 0.12a	0.02003	3.4 (0.9)
Arg	14.73 ± 0.09a	11.24 ± 0.18b	14.81 ± 0.11a	<0.0001	
Ala	4.74 ± 0.05a	3.31 ± 0.15b	4.29 ± 0.11a	0.01524	
Tyr	2.76 ± 0.22b	2.30 ± 0.13b	3.21 ± 0.09a	0.01387	
Cys	0.84 ± 0.06a	0.70 ± 0.12b	0.81 ± 0.15a	0.01376	
Val	4.18 ± 0.21b	3.50 ± 0.18c	4.62 ± 0.10a	0.02046	3.5 (1.3)
Met	1.16 ± 0.02b	0.99 ± 0.08b	1.44 ± 0.10a	0.02123	2.5 (1.7)
Phe	4.94 ± 0.05a	3.49 ± 0.07b	4.61 ± 0.12a	0.02312	6.3 (1.9)
Ile	3.28 ± 0.10b	3.03 ± 0.09b	3.99 ± 0.06a	0.04521	2.8 (1.3)
Leu	7.13 ± 0.07a	5.64 ± 0.12b	7.29 ± 0.08a	0.00311	6.6 (1.9)
Lys	2.58 ± 0.05a	2.01 ± 0.07b	2.13 ± 0.11b	0.03279	5.8 (1.6)
Pro	4.22 ± 0.07a	2.33 ± 0.04c	3.18 ± 0.01b	0.00323	

a All amino acid (AA) values are expressed as grams per 100 g of protein;

b Values are means ± SD of three determination, Mean values with different letters in the same row are significantly different (*P*-value <0.05);

c Numbers in parentheses of FAO/WHO recommended pattern (1990) represent essential amino acid for adults and numbers outside the parentheses represent essential amino acid for pre-school child (2~5 years).

**Table 3:** Some functional properties of DWF, WPC and WPI at their natural pH a.

Functional properties b	DFWF	WPC	WPI
EA (%)	53.28 ± 0.15 a	52.98 ± 0.43 b	50.01 ± 0.22 c
ES (%)	25.26 ± 0.27 c	27.45 ± 0.34 b	30.30 ± 0.25 a
FC (%)	24.35 ± 1.03 c	38.78 ± 2.23 b	46.34 ± 2.06 a
FS (%)	10.23 ± 2.15 c	28.18 ± 1.04 b	30.56 ± 2.35 a
FAC (g/g)	1.87 ± 0.20 c	2.50 ± 0.17 b	2.81 ± 0.09 a
WAC (g/g)	3.57 ± 0.36 a	2.94 ± 0.20 c	3.11 ± 0.28 b
Surface hydrophobicity (*H*o)	223.83 ± 5.87c	315.39 ± 6.54 a	276.51 ± 8.31 b

a Results represent the average of three determinations ± SD, values in the same row with different letters are significantly different (*p* < 0.05);

b WAC: water absorption capacity; FAC: fat absorption capacity; EA: emulsifying activity; ES: emulsion stability; FC: foam capacity; FS: foam stability.
